# Specialist perinatal palliative care: a retrospective review of antenatal referrals to a children’s palliative care service over 14 years

**DOI:** 10.1186/s12904-023-01302-5

**Published:** 2023-11-10

**Authors:** Sophie Bertaud, Georgina Brightley, Nicola Crowley, Finella Craig, Dominic Wilkinson

**Affiliations:** 1 Oxford Uehiro Centre for Practical Ethics, University of Oxford, Suite 8 Littlegate House, 16-17 St Ebbe’s Street, Oxford, OX1 1PT UK; 2 Louis Dundas Centre for Children’s Palliative Care, Great Ormond Street Hospital, London, UK; 3 Neonatal Unit, St George’s Hospital, London, UK

**Keywords:** Paediatric palliative care, Perinatal, Antenatal, Fetal

## Abstract

**Background:**

Perinatal palliative care is an emerging branch of children’s palliative care. This study sought to better understand the pattern of antenatal referrals and the role of a specialist paediatric palliative care (PPC) team in supporting families throughout the antenatal period.

**Methods:**

A single-centre retrospective chart review of all antenatal referrals to a quaternary children’s palliative care service over a 14-year period from 2007 to 2021.

**Results:**

One hundred fifty-nine antenatal referrals were made to the PPC team over a 14-year period, with increasing referrals over time. Referrals were made for a broad spectrum of diagnoses with cardiac conditions (29% of referrals) and Trisomy 18 (28% of referrals) being the most prevalent. 129 referrals had contact with the PPC team prior to birth and 60 had a personalised symptom management plan prepared for the baby prior to birth. Approximately one third (48/159) died in utero or were stillborn. Only a small number of babies died at home (*n* = 10) or in a hospice (*n* = 6) and the largest number died in hospital (*n* = 72). 30 (19% of all referrals) were still alive at the time of the study aged between 8 months and 8 years.

**Conclusions:**

Specialist PPC teams can play an important role in supporting families during the antenatal period following a diagnosis of a life-limiting fetal condition and demand for this service is increasing. A large proportion of the cases referred will not survive to the point of delivery and a number of babies may survive much longer than predicted. PPC teams can be particularly helpful navigating the uncertainty that exists in the antenatal period and ensuring that plans are made for the full spectrum of possible outcomes.

## Background

Since the world’s first children’s hospice opened in Oxfordshire in 1982, Paediatric Palliative Care (PPC) has rapidly become established as an important subspecialty within paediatrics, focusing on a multidimensional approach to the care of children and young people with life-limiting conditions [1]. Recent years have seen an expansion of PPC services into neonatal care [2] and the care of teenagers and young adults [3]. Extending palliative care into the antenatal period potentially represents the final frontier in the provision of children’s palliative care.

Increasingly sophisticated technology has enhanced our ability to accurately diagnose potentially life-limiting conditions during pregnancy. Improved prenatal screening coupled with increasing maternal age means that the fetal prevalence of conditions such as Trisomy 18 and 13 is increasing [4]. In countries where routine screening is in place, more than half of all congenital abnormalities are identified antenatally, including 74% of all major conditions [5]. Whilst a number of families opt for a termination of pregnancy following the diagnosis of a fetal anomaly [6], some elect to continue their pregnancy and providing adequate support to this cohort is important. Families who face the antenatal diagnosis of a life-limiting condition in their baby may be eligible for referral to a PPC service. However, we do not currently have an accurate picture of the number of these referrals and there is significant inequity in both the type and availability of PPC services across the UK, with a very small number of specialist PPC services and wide geographical variation in access to these services [7].

There have been a small number of studies worldwide reporting clinical experience of perinatal palliative care and only one published study from the UK [8–25], summarised in Table 1. This literature suggests that there is considerable variability in the fetal conditions referred to palliative care and in the support that is offered to families. For clinicians in fetal medicine there can be a lack of clarity as to which conditions warrant an antenatal palliative care consultation and when is the right time to refer to PPC services [26].

**Table 1:** Previous cohort studies of perinatal palliative care. Studies identified through a literature search across two databases (PubMed and CINAHL) using key words in the Title or Abstract

Authors	Year	Location	Study design	Number of cases	Cohort examined	Study period	2 most common diagnoses	Liveborn rate
Calhoun et al.	2003	Tacoma, Washington, USA	Descriptive study	33	Patients carrying a fetus with a clearly delineated lethal anomaly	Unspecified	Trisomy, Renal tract abnormality	61%
D’Almeida et al	2006	Illinois, USA	Descriptive study	28	Patients with a clearly defined lethal fetal anomaly eligible for perinatal hospice	3 years 6 months	Anencephaly, Trisomy	76%
Breeze et al.	2007	Cambridge, UK	Single centre prospective cohort	20	All pregnancies diagnosed with a lethal abnormality	4 years	Renal tract abnormality, Trisomy	30%
Marc-Aurele et al.	2013	San Diego, California, USA	Exploratory retrospective electronic chart review	66	All patients referred to a home perinatal palliative program	5 years 10 months	Trisomy, Anencephaly	62%
Bétrémieux et al	2016	Rennes, France	Retrospective study	20	Pregnancies with diagnosis of lethal fetal condition where parents accepted antenatally the proposal or sought for palliative care at birth	6 years	Congenital heart disease, CNS abnormality	95%
Kukora et al	2017	Ann Arbor, Michigan, USA	Single centre retrospective cohort	144	Patients referred for outpatient antenatal counselling by a neonatologist	2 years 6 months	Chromosomal (including Trisomy), Multiple anomalies	79%
Hostalery et al.	2017	Marseilles, France	Single centre retrospective cohort	39	A series of pregnancies with severe fetal abnormalities	10 years	Organ malformation only, Other pathology	64%
Bourdens et al.	2017	Southern France	Multi-centre retrospective cohort	155	Patients continuing pregnancies with a fetal pathology qualifying for a termination of pregnancy	9 years	Single organ malformation, Other pathology	79%
Marc-Aurele et al.	2018	San Diego, California, USA	Single centre retrospective cohort	332	Women diagnosed prenatally with a potentially life-limiting fetal diagnosis	6 years	Genetic defect, Trisomy	23%
Pfeifer et al	2018	Zurich, Switzerland	Single centre retrospective cohort	30	Patients prenatally assigned to palliative care	6 years	CNS abnormality, Chromosomal aberration	47%
McMahon et al	2018	Dublin, Ireland	Single centre retrospective cohort	83	Perinatal patients (antenatal and 6 weeks postnatal) referred to a specialist palliative care service	4 years	Trisomy, Congenital heart disease	55%
Kamrath et al.	2019	Minnesota, USA	Single centre retrospective cohort	27	Mother-infant pairs offered perinatal palliative care	3 years 9 months	Chromosomal abnormality, Skeletal dysplasia	(−)
Tucker et al	2021	Kansas City, Missouri, USA	Single centre retrospective cohort	430	Mothers met in the fetal health center whose infants were thought to have life-limiting conditions	9 years	Cardiac, Congenital anomaly	91%
Doherty et al	2021	Ottawa, Ontario, Canada	Single centre retrospective cohort	85	Infants with prenatally diagnosed life-limiting conditions that were referred for perinatal palliative care	10 years	Trisomy, Severe CNS malformation	66%
de Barbeyrac et al	2022	Paris Ile-de France region, France	Multicentre prospective observational study.	736^a^	Cases of prenatal diagnosis of a severe fetal condition considered eligible for TOP in which women opted to continue their pregnancy	2 years	Syndromic/polymalformative, CNS abnormality	72%
Buchholtz et al	2022	Berlin, Germany	Single centre retrospective cohort	118	Pregnant women and infants with potentially life-limiting conditions referred for prenatal palliative care counselling	4 years	Trisomy, Complex congenital condition	42%
Tewani et al	2022	Singapore, Singapore	Single centre prospective cohort	41	Cases referred to the perinatal palliative care service	3 years	Cranial malformation, Severe organ damage (heart, lung, kidney)	(−)
Buskmiller et al.	2022	Houston, Texas, USA	Single centre retrospective cohort	187	Perinatal palliative care consults for life-limiting anomalies	4 years	Cardiovascular/chest, CNS abnormality	80%

*TOP* Termination of Pregnancy, *CNS* Central Nervous System

^a^106 cases were identified as eligible for palliative care

We sought to review the experience at our centre over the last 14 years in order to better understand the pattern of antenatal referrals and the role that a specialist PPC team can play in supporting families in the antenatal period.

## Methods

Antenatal referrals to the PPC team between 2007 and 2021 were identified using internal electronic database information. The Louis Dundas Centre for Children’s Palliative Care (LDC) at Great Ormond Street Hospital in London is a quaternary level specialist multidisciplinary PPC service. The LDC receives approximately 250 referrals every year from Greater London and South East England. Antenatal referrals come from 12 fetal medicine and obstetric centres across the region. There is no maternity unit at GOSH and all referrals are out-born. Antenatal support is usually offered by the PPC team via attendance at antenatal appointments and multidisciplinary meetings at other centres and via telephone consultations and home visits. The PPC team will typically work alongside local teams to make plans for the baby’s birth and postnatal care and this may involve preparing a Symptom Management Plan (SMP). Families are offered holistic support and we will usually involve the family’s local children’s hospice for additional support.

A retrospective review of both the maternal and, where relevant, child’s medical notes was carried out by three clinicians using a standardised electronic abstraction form with cross-verification to validate the data. We collected data on gestation at referral, fetal diagnosis, maternal ethnicity and religion, number and type of encounters with PPC, whether the family were previously known to PPC, whether a SMP was prepared prior to birth, date and location of death if applicable, whether a bereavement appointment was offered and age of surviving children (at time of final data collection in December 2022). Any discrepancies in data coding were reviewed jointly and discussed. Only data that had previously been collected as part of clinical care was extracted from the medical notes and only members of the patient’s direct clinical care team had access to the medical records. Data was anonymised with each patient being allocated a unique study code.

NHS Research Ethics Committee approval was not required for this study. Approvals were obtained from the Great Ormond Street Hospital Information Governance Team.

## Results

**Fig. 1:**
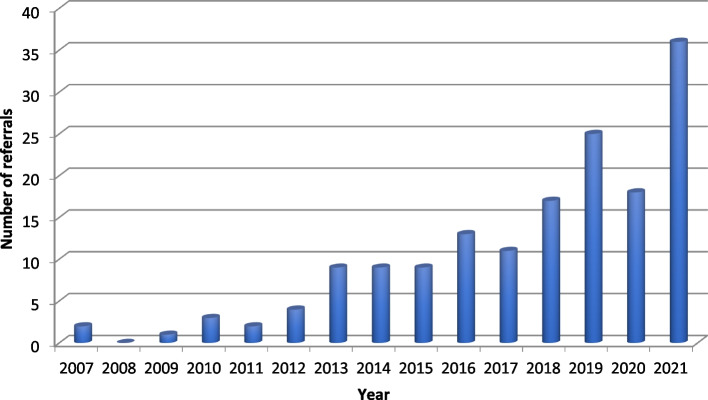
Number of antenatal referrals to the specialist PPC team by year

One hundred fifty-nine antenatal referrals were received over the study period. Figure 1 illustrates a pattern of increasing referrals over time from 2 per year in 2007 to 36 per year in 2021.

### Timing of referrals

The largest number of referrals were between 21 and 30 weeks gestation (74/159, 47%), followed by referrals between 30 and 35 weeks (54/159, 34%). A smaller proportion of patients were referred late (after 35 weeks, 25/159, 16%) or early (between 12 and 20 weeks, 5/159, 3%).

Four of the 159 families were previously known to the PPC team, due to having a previous child or children with a life-limiting condition.

### Referral type

Referrals were made for a broad spectrum of fetal diagnoses with cardiac conditions (29% of all referrals) and Trisomy 18 (28% of all referrals) being the most prevalent. Table 2 illustrates the categories of fetal diagnoses for which antenatal referrals were made.

**Table 2:** Diagnostic categories of antenatal referrals to a specialist PPC team

Diagnostic category	Number of referrals received during study period
Cardiac disorders	46
Trisomy 18	44
Anencephaly/Holoprosencephaly/Hydranencephaly	13
Abnormal brain/spinal cord development	13
Renal disorders	12
Trisomy 13	11
Other genetic disorders	10
Miscellaneouss	5
Skeletal dysplasia	2
Conjoined twins	2
Severe hydrops fetalis	1
**TOTAL**	**159**

### Contact with the PPC team

30/159 patients (19%) were referred but did not meet the PPC team (largely because of late referral, see discussion). The number of encounters for the remainder was 1 to 2 in 62% (98/159), 3 to 4 in 13% (21/159) and over 4 in 6% (10/159). Of the 129 families met by PPC prior to delivery, 87 had only face-to-face consultations, 35 had a mix of face-to-face and telephone consultations and 7 families had encounters by telephone only. 60 out of the 159 cases had a personalised SMP prepared for the baby prior to birth with guidance on how to manage potential symptoms using both pharmacological and non-pharmacological methods.

### Age at death

**Fig. 2:**
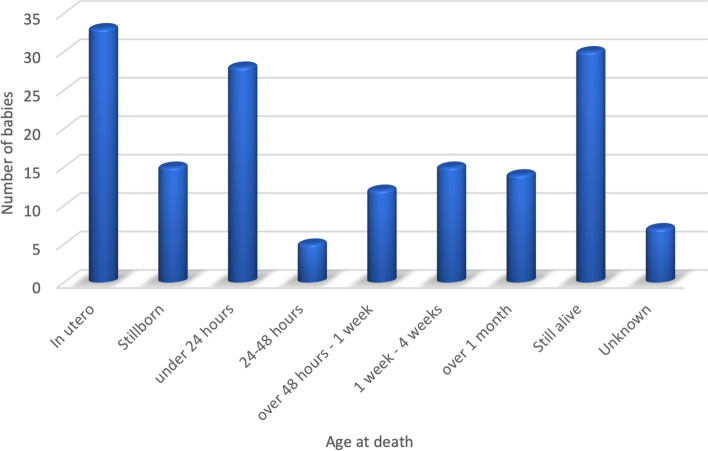
Age at death for babies referred antenatally to the PPC team

Figure 2 illustrates the age at death for the babies referred antenatally to the service. Of the 159 cases, 33 died in utero, 15 were stillborn, 28 died under 24 hours of age, 5 died between 24 and 48 hours, 12 died between 48 hours and 1 week of life, 15 died between 1 week and 4 weeks, 14 died at over 1 month old (5 with a diagnosis of Trisomy 18, 3 with cardiac disorders, 3 with abnormal brain/spinal cord development, 2 with holoprosencephaly and 1 with Trisomy 13), 30 were still alive at the time of the study and for 7 patients this data was missing. Table 3 illustrates the diagnoses and age at time of study for the 30 surviving children.

**Table 3:** Characteristics of surviving children (at the time of the study) for babies referred antenatally to the PPC team

Diagnosis	Number of cases	Age or age range for children at the time of the study
HLHS	8	8 m to 8y 10 m
Holoprosencephaly	3	1y to 4y 6 m
Dandy-Walker malformation	3	11 m to 4y
Complex congenital heart disease	3	10 m to 1y 3 m
Tricuspid atresia with VSD	2	11 m to 1y 6 m
Trisomy 18	1	8y 2 m
Spina bifida	1	8y 1 m
Arthrogryposis	1	5y 3 m
Microcephaly	1	2y 4 m
Epstein’s anomaly	1	2y 1 m
Unbalanced AVSD	1	1y 11 m
Hypoplastic right heart syndrome	1	1y 10 m
Suspected coarctation of the aorta, severe ventriculomegaly	1	1y 9 m
Pulmonary atresia	1	1y 1 m
Multiple congenital abnormalities including renal and gut issues	1	1y
Double inlet left ventricle	1	1y

*HLHS* Hypoplastic Left Heart Syndrome, *VSD* Ventricular Septal Defect, *AVSD* Atrioventricular Septal Defect, *y* years, *m* months

### Place of death

Place of death was as follows (this excludes the 7 patients where age at death was unknown, 33 in utero deaths and 30 patients who were still alive): home (10/89), hospice (6/89), birth hospital (55/89), hospital transferred to (17/89) and unknown (1/89). Of the 10 children who died at home, 6 were over 1 month old when they died (age range 2 months to 6 years), 3 were between 1 week and 4 weeks old and 1 baby was over 48 hours old but less than 1 week old.

## Discussion

A specialist PPC team can provide support to families navigating the challenging uncertainty of a life-threatening antenatal diagnosis, enabling them to make plans in anticipation of a range of possible outcomes. Parents who have received antenatal palliative care report positive experiences and describe the value of compassion from healthcare professionals, their babies being treated ‘as a person and not as a diagnosis’ and finding ways to honour their babies [27–29]. For some families the time they get to spend with their baby is very short, and extremely precious, and perinatal palliative care can help to ensure that families have no regrets about how they spend that time [30].

In this retrospective review we have demonstrated a striking increase in the number of referrals to PPC over the last 14 years from 1 to 2 per year to more than 30 per year. This likely reflects an increasing awareness of the role for specialist perinatal palliative care support [31]. The overall number of referrals, however, remains small and potentially represents only a small fraction of the eligible families. We do not have accurate data for the number of life-limiting conditions diagnosed in pregnancy each year in the UK but we do know that the live birth rate for London is approximately 120,000 live births each year [32] and that the birth prevalence for the 11 auditable conditions screened under the Fetal Anomaly Screening Programme (including anencephaly, HLHS, bilateral renal agenesis, lethal skeletal dysplasias and Trisomy 13 & 18) is 77 per 10,000 total births (live births and stillbirths) for London and the South East [6]. Even with conservative estimates this suggests that the number of life-limiting antenatal diagnoses each year in our referral region is likely to be in the hundreds. It may be that a number of these families are supported locally by fetal medicine and neonatal teams. Whilst we do not have this data for the UK context, a French study of prenatal decision-making processes found that it was very rare to have a palliative care specialist present in prenatal discussions (this occurred in only 2.8% of cases where perinatal palliative care was considered) [22].

The largest number of referrals came in the period between 21 and 30 weeks. This is likely to represent the fact that a number of congenital anomalies are first suspected at the fetal anomaly scan, which takes place at around 20 weeks gestation [6]. There were however a significant number of referrals in later gestation; 79 of the 159 referrals were received after 30 weeks. In practice referral to PPC usually only occurs if a family has elected to continue with the pregnancy, but there may arguably be a role for PPC support whilst parents remain in a decision-making phase or where there remains uncertainty around the baby’s prognosis. The significant variability observed in timing of referrals, and whether patients are referred at all, may indicate a need for clearer guidance.

As one of the largest published cohorts of antenatal referrals to PPC, the spectrum of conditions referred is also potentially informative. For example, we found a large number of referrals for fetuses with cardiac disease in our cohort (*n* = 46). From 2020 onwards it has been the policy of our fetal cardiology team to refer all single ventricle patients to PPC. However even prior to this policy, the majority of referrals involved diagnoses of congenital heart disease or cardiac anomalies associated with features of Trisomy 13 or Trisomy 18, all of which are reviewed in fetal cardiology clinic. Our population may be somewhat unique given the presence of three paediatric cardiac centres in the London region, but the large number of antenatal referrals with cardiac disease nevertheless points to an important role for joint working between fetal cardiology and PPC teams in the future.

Within this study, 30 of the referrals did not meet the PPC team before birth and in most cases, this was because the baby either died in utero or the referral was received late in pregnancy and the baby delivered before a first meeting could be arranged. In 2 cases, it was documented that the family declined antenatal input from PPC. This highlights the importance of equipping all healthcare professionals working in antenatal care with foundational knowledge in palliative care and the communication skills necessary to handle these sensitive consultations.

Approximately half of the cases who were met by the PPC team had a personalised SMP prepared (60/129) for the baby before birth which gave guidance on how to manage potentially distressing symptoms such as pain, breathlessness and excessive secretions. These plans include suggestions for symptom management using non-pharmacological techniques as well as recommended doses for medications on a dose per kg basis until a birth weight is available. Our anecdotal experience is that medical teams value having access to SMPs in advance of a baby’s birth and that they can be a useful basis for discussion with parents around what symptoms they may expect to see in their baby. There is however an important lack of data in the literature around the prevalence of symptoms early after delivery and the value of SMPs in perinatal palliative care, which warrants further attention.

Only a very small number of babies in this cohort died at home (*n* = 10) or at a hospice (*n* = 6) and the largest number of babies died in hospital (*n* = 72). Those children who died at home tended to be older, with no babies who died at less than 48 hours old making it home. Whilst we do not have data on parental preference for place of death for these cases, there are recognised barriers to both offering and achieving a choice of place of death for seriously ill neonates [33]. Further research would be helpful to address whether choice of place of death could be more readily explored during the advance care planning process. In our study 28/159 babies died within 24 hours suggesting that in specific cases where the risk of early postnatal demise is particularly high, parental counselling around preferred place of death may need to take this into account.

A large number of babies (48/159) in our cohort died either in utero or during delivery which is reflective of the liveborn rate observed in previous studies (Table 1). Not knowing whether a fetus will survive to birth or not can be immensely difficult for both families and health care professionals and the palliative care team may play a role in navigating this space of uncertainty. The process of planning a baby’s management after birth can be supportive and therapeutic in and of itself, even if the baby does not survive to the point of delivery [34].

There was a large group of surviving babies in our cohort (*n* = 30). Aside from one child with Trisomy 18 who was 8 years old at the time of this study and one child with arthrogryposis (5 years old) these surviving babies all fell into two categories; either congenital heart disease (including Hypoplastic Left Heart Syndrome) or severe Central Nervous System abnormalities (including holoprosencephaly). This points to the important role that palliative care teams can play in ensuring that parallel planning takes place for these families. Parallel planning - planning for life while also planning for deterioration or death [35] - allows families to be prepared for the worst possible outcome but also simultaneously counselled about plans for if their baby survives. Indeed, it can sometimes be incredibly difficult for families to adjust to the reality of bringing their baby home if they have not been told about the possibility of their baby surviving. In these cases, it is also vital that appropriate expertise is sought from the relevant specialists, such as fetal cardiologists and neurosurgeons. For some of these children, palliative care will be offered alongside curative treatment, or treatment aimed at significantly prolonging life.

Antenatal counselling and support is provided by large multi-disciplinary teams, which include midwives, fetal medicine specialists, obstetricians, neonatologists and paediatric sub-specialists, and equipping all members of the team with the confidence to deliver elements of PPC is important. Professionals working in fetal medicine and paediatrics have themselves identified the need for integrated PPC services that take a multi-professional approach and which incorporate education and training for all health care professionals involved [36, 37].

Finally, as prenatal imaging techniques and genetic screening continue to improve, establishing robust provision of antenatal palliative care will become ever more important as more families face increasingly complex decisions. Importantly, NICE identified perinatal palliative care as one of five key research recommendations in their 2016 guideline on ‘End of life care for infants, children and young people with life-limiting conditions’ [38]. There is a need for further prospective research looking at the experience of families following the diagnosis of a life-limiting condition during pregnancy, the support they are currently offered and the role that PPC services can play in this support.

### Study limitations

This was a retrospective chart review and was therefore reliant on the accuracy of the data available in the medical notes. We attempted to look at other features of the antenatal referrals including parental ethnicity and religion and bereavement follow-up but unfortunately a lot of this data was missing, making it too incomplete to usefully interpret. We have also not captured here those babies diagnosed with a life-limiting condition antenatally but who were not referred to the PPC team until after birth. This study also lacks any qualitative assessment of the experience of parents (as well as that of health care professionals), which is crucial in understanding the impact of the support offered to families by the PPC team.

## Conclusions

Specialist PPC teams can play an important role in supporting families during the antenatal period following a diagnosis of a life-limiting fetal condition and demand for this service is increasing. The range of outcomes for the babies illustrated in this study underlines the uncertainty that exists for families in the antenatal period and identifies a crucial role for palliative care teams in facilitating meaningful parallel planning in conjunction with the appropriate disease specialists.

## Data Availability

The datasets used and/or analysed during the current study are available from the corresponding author on reasonable request.

## Abbreviations

AVSD: Atrioventricular septal defect
CNS: Central nervous system
HLHS: Hypoplastic left heart syndrome
LDC: Louis dundas centre for children’s palliative care
PPC: Paediatric palliative care
SMP: Symptom management plan
TOP: Termination of pregnancy
VSD: Ventricular septal defect
